# Challenges for Complex Microbial Ecosystems: Combination of Experimental Approaches with Mathematical Modeling

**DOI:** 10.1264/jsme2.ME13034

**Published:** 2013-08-30

**Authors:** Shin Haruta, Takehito Yoshida, Yoshiteru Aoi, Kunihiko Kaneko, Hiroyuki Futamata

**Affiliations:** 1 Department of Biological Sciences, Graduate School of Science and Engineering, Tokyo Metropolitan University, 1–1 Minami-Osawa, Hachioji, Tokyo 192–0397, Japan; 2 Department of General Systems Studies, University of Tokyo 3–8–1 Komaba, Meguro, Tokyo 153–8902, Japan; 3 Institute for Sustainable Sciences and Development, Hiroshima University, 2–113 Kagamiyama, Higashi-Hiroshima, Hiroshima 739–8527, Japan; 4 Department of Biology, Northeastern University, 360 Huntington Ave. Mugar Lifescience Bldg., Boston, MA 02115, USA; 5 Research Center for Complex Systems Biology, University of Tokyo, Meguro-ku, Tokyo, 153–8902, Japan; 6 Department of Applied Chemistry and Biochemical Engineering, Graduate School of Engineering, Shizuoka University, Hamamatsu, 432–8561, Japan

**Keywords:** complex systems, mathematical modeling, plasticity, interaction, diversification

## Abstract

In the past couple of decades, molecular ecological techniques have been developed to elucidate microbial diversity and distribution in microbial ecosystems. Currently, modern techniques, represented by meta-omics and single cell observations, are revealing the incredible complexity of microbial ecosystems and the large degree of phenotypic variation. These studies propound that microbiological techniques are insufficient to untangle the complex microbial network. This minireview introduces the application of advanced mathematical approaches in combination with microbiological experiments to microbial ecological studies. These combinational approaches have successfully elucidated novel microbial behaviors that had not been recognized previously. Furthermore, the theoretical perspective also provides an understanding of the plasticity, robustness and stability of complex microbial ecosystems in nature.

The complexity of an ecosystem was well recognized by Charles Darwin, who described the biosphere in the words “tangled bank” (7). Application of molecular techniques to microbial ecology is revealing the incredible diversity and variety of interspecies relationships in the microbial world. These achievements demonstrate that micro-environments are markedly complex systems. However, even a model ecosystem composed of three species has been shown to be considerably complex. As a representative example describing the three-species relationships, Paine reported that selective predation by a sea star *Pisaster* maintained the coexistence of two species of shellfish that competed with each other in a bay (48). A bacteriological study demonstrated the complexity of bacterial relationships as being similar to rock-paper-scissors among three strains, *i.e.*, a colisin-producing strain, a colisin-resistant strain and a colisin-sensitive strain (37). A series of recent studies attempted to assess network relationships among members in a stable mixed culture system composed of four bacterial species (21, 34, 35, 70). These studies indicated that conventional experimental researches should be complemented by other approaches to comprehend complex ecosystems.

Mathematical modeling has been traditionally applied to predict population dynamics and to evaluate the carrying capacity of environments since the 19th century. Consequently, microbial growth has been well documented by logistic curves. The Lotka-Volterra equations proposed in the 20th century are widely utilized to describe the population dynamics of ecosystems containing competitive interactions or a predator-prey system. Currently, progress in computers is allowing mathematical analyses to deal with a variety of complex biological behaviors, such as population dynamics, circadian rhythm, morphogenesis, genetic evolution and immune system. Mathematical approaches will potentially help to understand ecosystems, to predict future behaviors and to propose new hypotheses through model construction and model analysis on the basis of experimental data. Mathematical interpretation will help to develop a microbial ecological theory.

We review the application of advanced mathematical approaches to microbial ecological studies and provide a theoretical perspective. “*Feedback between ecological and evolutionary dynamics: experimental study using planktonic microorganisms*” introduces a series of studies on a predator-prey system by laboratory experiments and mathematical modeling to elucidate the adaptive changes in microbial phenotypes that drive population dynamics. “*Trends in understanding the dynamics of microbial ecosystem*” describes an example applying kinetic modeling to a chemostat culture. Mathematical modeling using certain parameters is ineffective to elucidate microbial behavior not previously recognized. “*In silico biofilm as a tool for investigation of microbial eco-system*” overviews combinational approaches of laboratory experiments and *in silico* analyses by reviewing studies on multispecies biofilm. Finally, “*Complex-systems biology for plasticity-diversity problem in ecosystem*” proposes practical approaches to understand the plasticity, robustness and stability of complex microbial ecosystems from a theoretical point of view.

It is our hope that this review will enhance communication among microbiologists, ecologists and mathematicians for mutual understanding to develop new concepts in their study fields.

## Feedback between ecological and evolutionary dynamics: experimental study using planktonic microorganisms

Understanding the pattern of temporal changes in organism abundance has been attracting the attention of ecologists although, due to various aspects of research capacity and technical difficulties, our understanding of population dynamics is still limited, especially for wild populations. For example, Kendall *et al.* (36) conducted a meta-analysis to identify the qualitative nature of dynamics in wild populations, and found that about one third of wild populations fluctuate in abundance and the remaining two thirds are stable. We know very little about how these qualitatively different population dynamics are shaped in nature. Understanding population dynamics involves three technical steps (62): [1] describing the pattern of population dynamics by observation studies, [2] finding the mechanisms that drive the population dynamics by, for example, experimental studies, and [3] formalizing the mechanisms into a mathematical model and reproduce the observed pattern by the model. It is easy to imagine the difficulty of performing all three steps, and indeed we do not have many examples of research that successfully explained population dynamics in nature. Studying the dynamics of populations reared in laboratory conditions can provide the principles of population dynamics, which will hopefully help to understand population dynamics in nature. Yoshida *et al.* have been using planktonic microorganisms cultured in the laboratory as a model for studies of population dynamics.

The conventional analysis of population dynamics assumes that organisms have fixed traits or a constant phenotype so that the strength of interactions between species does not change over time. For example, different prey species could have a different extent of defense against a specific predator, resulting in different strengths of the predator-prey interaction depending on the specific predator and prey pair, but this interaction strength would not change and would be constant for the pair. However, evolutionary ecological studies have shown that organisms can indeed change their phenotype traits in response to changes in ecological and environmental conditions (59). Organisms have the ability to adaptively change their phenotype by means of rapid microevolution using genetic diversity within a population or phenotypic plasticity without changing the genetic structure of a population. The adaptive change of a trait can result in a change of interaction strength if the trait is associated with the interaction, which suggests that we need to understand how the adaptive change of traits can alter the pattern of population dynamics.

We have shown the influence of adaptive change on the population dynamics by laboratory experiments using planktonic microorganisms. Our study system is a predator-prey system that consists of an algal prey (*Chlorella vulgaris*) and a rotifer predator (*Brachionus calyciflorus*), reared in a chemostat culture. *C. vulgaris* reproduces only asexually so that a population is a collection of clones. *B. calyciflorus* can reproduce asexually and sexually, but our laboratory population evolved to reproduce only asexually, because the turbulent condition in the chemostats prevented mating between males and females (14).

We found that our algal population had genetic diversity with regard to defense against rotifer predation (72). This defense is associated with competitive ability to obtain a limited nutrient (*i.e.* nitrate in our system) and there is a tradeoff relationship between defense and competitive ability. A defended clone is advantageous when rotifer predators are abundant, whereas an undefended clone is advantageous when predators are scarce and competition for the limited nutrient is severe. This genetic diversity of the algal population was a key determinant of population dynamics of the algal and predator populations (71). By manipulating algal genetic diversity, we were able to show qualitatively different population cycles of the predator-prey system. If the algal population consisted of a single clone and thus there was no genetic diversity, *i.e.*, the raw material for evolutionary changes, the predator-prey system showed shorter population cycles (*ca.* 7 to 10 days) with regular phase lag between predator and prey cycles as in the Lotka-Volterra predator-prey model. On the other hand, if the algal population consisted of multiple clones that had a tradeoff between defense and competitive ability and thus there was genetic diversity that allowed evolutionary responses, the system showed longer population cycles (*ca.* 30 to 60 days) with the unusual, out-of-phase lag between predator and prey cycles. Thus, the algal genetic diversity markedly altered the pattern of population dynamics of the algal-rotifer system.

The evolutionary change of the algal population was revealed by a newly developed molecular method that can quantify the relative frequency of a pair of different clones (43). When the rotifer predator was in low abundance and algal abundance was high, which implies severe competition for the limited nutrient, the undefended clone with superior competitive ability was selected for and increased in frequency within the algal population. When the rotifers increased their abundance and total algal abundance declined, the defended clone with inferior competitive ability was selected for and was eventually fixed in the population because rotifer abundance was kept high for the rest of the experiment. This study clearly showed the evolutionary change of the algal population that has been thought to occur and that produced a change of population dynamics in the above-mentioned experiment (71).

The evolutionary change of the algal population also produced other qualitatively different population dynamics (73). In regular population cycles, an increase of predator abundance should correspond to a decrease of prey abundance or vice versa, although there is often a time lag in the response. Our predator-prey system showed that algal abundance was almost constant whereas rotifer abundance fluctuated greatly, so that prey abundance did not respond to the increase or decrease of predator abundance. If we did not have prior information that the rotifer was an actual predator of the alga and there was no other organism contained in the chemostats, we would have concluded that there was no predator-prey relationship between the rotifer and the alga because of the apparent non-response of the alga to the rotifer predator. Our mathematical model showed that the evolutionary change of the algal population can produce a strange pattern of population dynamics if defended and undefended algal clones compensate for the change of rotifer density with the decrease of one type well balanced by the increase of another type.

**Fig. 1:**
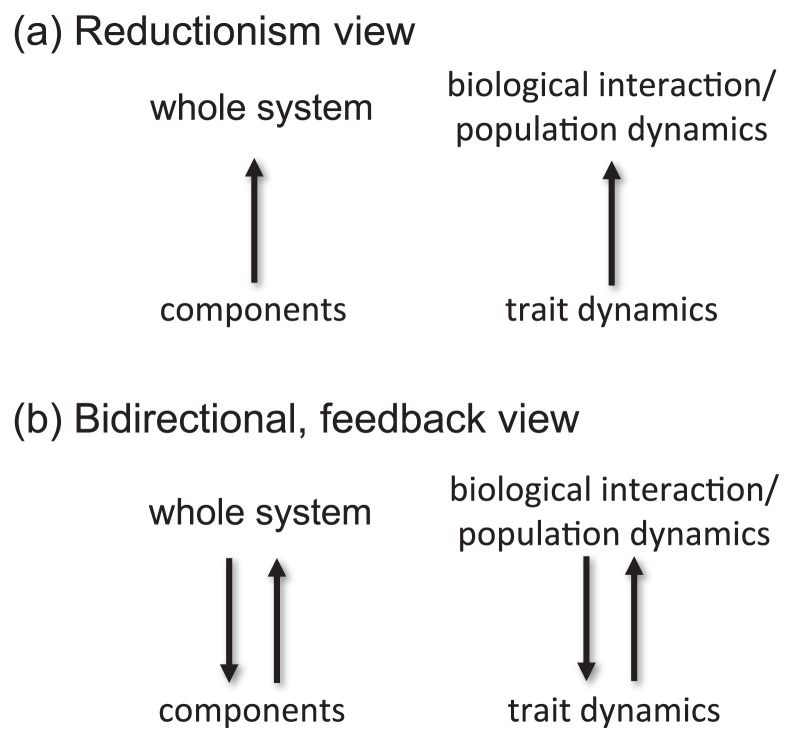
Simplified schemes of the reductionism view (a) and bidirectional, feedback view (b). In biological communities such as predator-prey systems, the reductionism view provides unidirectional cause-effect relationships, for example, between traits of interacting species and the nature of interaction/population dynamics driven by the interaction. In reality, however, the relationship between the two levels (components and the whole system) tends to be bidirectional and to involve feedback. For example, trait changes can influence the interaction and population dynamics that in turn shape how the trait changes.

The genetic diversity and consequent evolutionary changes of the algal population altered the population dynamics of the algal-rotifer system markedly. The change of rotifer density produced selection pressure on the algal population that responded evolutionarily using genetic diversity. Thus, there was a tight interaction between the trait-level dynamics (*i.e.* evolutionary change) and the population-level dynamics (*i.e.* population cycles). Changes in one level resulted in the response of the other level, which produced a feedback to the response of the original level. Recognizing the feedback relationship among different biological levels was necessary to understand our algal-rotifer system with regard to dynamics at trait and population levels. This understanding clearly needs insight beyond reductionism and we could not accurately understand the predator-prey system if we looked at the hierarchic system unidirectionally (Fig. 1).

Although we observed feedback between different biological levels (*i.e.* phenotype and population levels) in our simple experimental system, which consisted of only a pair of interacting species, whether such feedback actually works in a wild community that has more interacting species is obviously the next question to address. If adaptive changes of one species somehow result in alterations of the biological community or environmental conditions, and if the altered community or environment possesses new selection pressure that feeds back to the original species and leads to adaptation of the species, there will be feedback among the different biological levels (56). Some recent studies showed part of this feedback relationship, and only a few studies have elucidated the whole picture of the feedback within a single system (13, 17). Thus, we are still far from concluding that the feedback among different biological levels is universally important in wild systems. This is where laboratory experiments can contribute by elucidating whether and how feedback works in different contexts and in different types of biological communities. For example, compared to the feedback in predator-prey systems, our understanding is very limited in competitive systems where adaptation of competitors may have an influence on the competitive outcome of interacting species. Experimental studies using microorganisms are a promising approach because adaptive responses of microorganisms tend to be rapid and more likely result in changes in population abundance, either of which makes it easier to examine the dynamic nature of the feedback.

## Trends in understanding the dynamics of microbial ecosystem

It is a great challenge for microbial ecologists to understand and control microbial ecosystems, which is also desirable for efficient bioremediation, wastewater treatment, agriculture field, human health, and etc. Microbial populations affect each other and build up their ecosystem. The microbial ecosystem is affected by its surrounding environment, and vice versa (10, 22). Although the bacterial community is constructed under environmental conditions (self-organization), the community structure is not stable, but fluctuates (bacterial community succession). It is predicted that the stability/sustainability of an ecosystem is maintained by the dynamics of the bacterial community structure (dynamic equilibrium mechanism). However, we do not know the basis of the self-organization and dynamic equilibrium mechanism.

It has been reported that seasonal marine bacterial succession in the community composition was robust (18). What is the driving force of community succession and its robust seasonal cyclicity? By analyzing the relationships among bacteria, eukaryotes, and environmental factors, Gilvert *et al.* suggested that interactions were strongest within domains of bacteria and eukaryotes rather than between them, and correlated relationships were stronger between taxa than between environmental variables. This may indicate that biological rather than physicochemical factors may be more important in defining the fine-gain community structure. It is suggested that robust seasonal cyclicity is also self-evident in the interactions between members of the community.

**Fig. 2:**
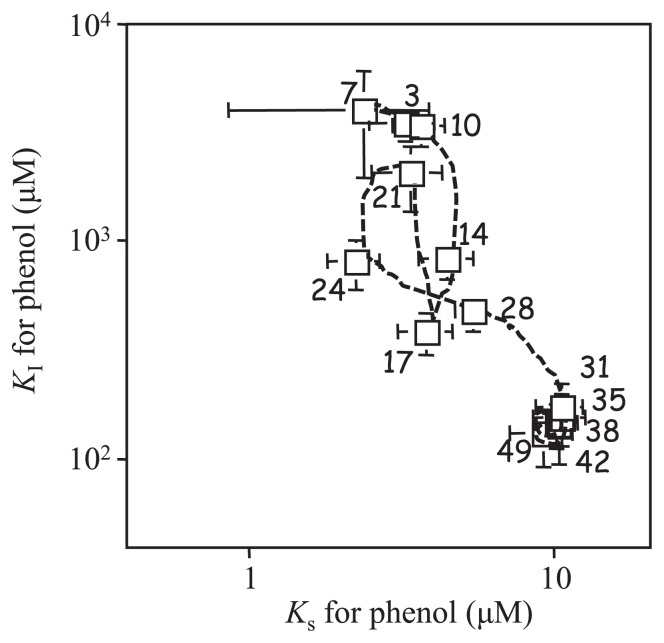
Kinetic parameters for phenol of chemostat bioreactor. Number means the sampling date.

**Fig. 3:**
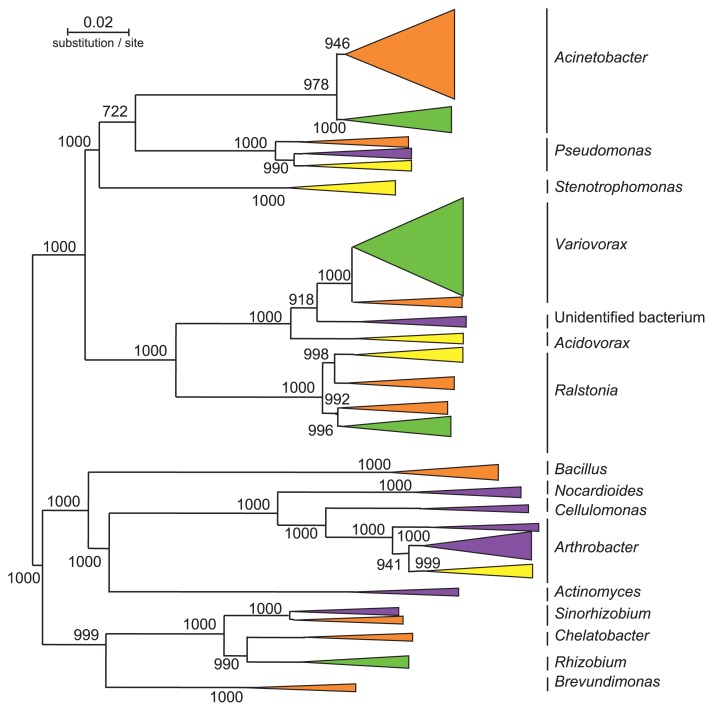
An unrooted neighbor-joining tree based on the nucleotide sequences of 16S rRNA genes from strains isolated from chemostat bioreactor. Purple, yellow, orange and green clusters indicate the clusters of strains isolated on day 0, day 3, day 10 and day 32, respectively. Numbers at the branch nodes are bootstrap values (per 1,000 trials); only values >500 are indicated. Bar represents 0.02 substitutions per site.

**Fig. 4:**
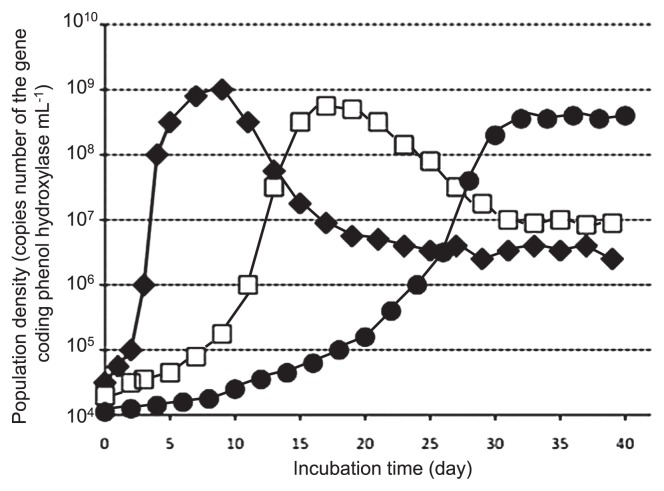
Image of bacterial community succession. For example, filled diamonds: first dominant (*Pseudomonas*), open squares: second dominant (*Acinetobacter* or *Ralstonia*), filled circulars: third dominant (*Variovorax*). Practical population densities of *Pseudomonas, Acinetobacter* or *Ralstonia* in the chemostat bioreactor are unknown but that of *Variovorax* is shown (17).

Laboratory-based microbial model systems have been shown to be useful in addressing ecological questions (24). As a model microbial ecosystem, a chemostat bioreactor was constructed with aquifer soil as the inoculum and phenol as the sole carbon and energy source, resulting in an enrichment culture of soil-bone phenol-degrading bacterial communities (15, 16). Kinetic parameters, *K*_S_ and *K*_I_ values, for phenol in the chemostat culture were analyzed according to the method described previously (15, 65) (Fig. 2). *K*_S_ is the half-saturation constant and *K*_I_ is the inhibition constant. These *K*_S_ and *K*_I_ values were stable at 3.1±0.65 μM and 3,600±290 μM, respectively, until day 10. Interestingly, these parameters oscillated; in particular, *K*_I_ values changed between 3,400 μM to 170 μM for the next 20 days. Finally, *K*_S_ and *K*_I_ values were stable at 10.1±0.51 μM and 150±17 μM, respectively. These parameters oscillated from the position of low *K*_S_ and high *K*_I_ to that of high *K*_S_ and low *K*_I_, although the *K*_S_ value is usually stable at a lower level in a chemostat culture. Bacterial community structure was analyzed by culture-dependent and -independent techniques. Phylogenetic analysis targeting 16S rRNA genes of strains isolated from the chemostat culture revealed that bacterial community succession occurred (Fig. 3), *e.g.*, *Pseudomonas* and *Arthrobacter* genera were initially the dominant bacteria and then the *Acinetobacter* genus became dominant from day 10, while the *Variovorax* genus, which was initially one of the minor populations, became dominant after day 32 (Fig. 4). Intra-genus succession was also observed in the *Acinetobacter*, *Pseudomonas*, *Variovorax* and *Ralstonia* genera. DGGE analyses also showed bacterial community succession (16). These results revealed that the dynamic change of the microbial ecosystem occurs at both community and intra-genus levels. Furthermore, it was indicated that the kinetic parameters for phenol of the *Variovorax* strains corresponded to those of the bioreactor on day 31 to day 49 (16). These results suggest the following: [i] the initially most abundant bacteria do not maintain their dominance; in parallel, a minor population then becomes dominant, [ii] however, the function of the system (complete degradation of phenol) is maintained, resulting in a sustainable ecosystem. Although the mechanism for complex bacterial community succession is not known yet, it was demonstrated that the *λ* value is a useful parameter for predicting a dominant strain in a two-strain mixed culture (21). The *λ* value is calculated according to the formula; *λ* =(*K*_S_×*D*)/(μ−*D*), where *K*_S_ is a half-saturation constant (mg L^−1^), *D* is the dilution rate (h^−1^), and μ is the growth rate constant (h^−1^). This means that a strain exhibiting a lower *λ* value will become dominant in a two-strain mixed culture. However there is need to investigate whether the *λ* value theory is valid for a mixed culture of several strains. Furthermore, the properties of a given bacterium as a substrate are not sufficient for understanding the complex system, and several analyses based on population-population interactions may be needed to understand the ecosystem network, including the metabolic process.

## *In silico* biofilm as a tool for investigation of microbial eco-system

### General introduction to mathematical modeling

Recently, mathematical modeling together with computer simulation, the so-called “*in silico*” approach, has come to the attention of the field of biology. In this section, we discuss the potential and challenges of the “*in silico*” approach as a tool for investigating the microbial eco-system for better understanding of the system, focusing on creating virtual biofilm as an example.

In general, mathematical modeling of a natural system is the process of creating a mathematical representation of a natural phenomenon and attempting to match experimental observation with symbolic statement. Mathematical modeling in the science and engineering field is used generally for the following two purposes: “Understanding” and “Prediction” of phenomena in natural or engineered systems. The main engineering objective of modeling is the “prediction” of processes to be investigated and controlled. On the other hand, the scientific objective of modeling is “integrative understanding” of the system to be investigated by verifying hypotheses because modeling provides an explanation of the system from a more theoretical point of view. The advantages of *in silico* experiments are well known as follows. First, they are usually much cheaper and faster than a laboratory experiment and thus can be repeated easily. Second, the experiments can be conducted under ideal conditions and are not subject to disturbing external influences. Third, an interesting process can be isolated easily. Fourth, the system behavior can be investigated under extreme conditions, which are often difficult to generate in a laboratory experiment. Beyond these advantages, constructing an artificial natural system and comparing these data with the real system can be a process in the integrated understanding of the complex natural system.

### Modeling of biofilm

Biofilm developed in various natural environments is a dynamic and highly complex system, composed of multispecies microorganisms (8). Their growth is characterized by complex three-dimensional structures, including channels, voids, towers, and mushroom-like protrusions, and their changing characteristics in response to environmental conditions (60). In this decade, there have been marked advances in experimental techniques for the *in situ* identification of microbial and physical structures of biofilm. Combined use of several techniques, such as fluorescent *in situ* hybridization, autoradiography techniques, and micro-sensors, enables *in situ* identification of microbial types, functions, and their activities at a single cell level (46, 58). Furthermore, having been recently developed and widely spreading in the field of microbial ecology, omics approaches will provide huge amount of data on individual components of the biofilm system (46).

Modeling a biofilm system describes mathematically the structure and activity of biofilm and to be able to dynamically represent a biofilm structure from the initial environmental conditions. Modeling of biofilm represents [i] heterogeneous morphology, [ii] spatial distribution of multiple species of microbial cells and their activities resulting from cell growth and decay, [iii] production of extracellular polymetric substances (EPS) and their distribution, [iv] spatial distribution of multiple soluble substances resulting from consumption and production by metabolic activity, and transportation by diffusion and convection, [v] hydrodynamics that affect mass transport efficiency and the physical structure of the biofilm, [vi] concentration of bulk liquid phase resulting from the above phenomena (8).

### Stochastic discrete model

To capture such a complex structure of biofilm, stochastic multi-dimensional, and bottom-up type models have been developed, such as grid-based modeling, commonly know as CA (cellular automata) (5, 45, 54), continuum type modeling (2), individual-based modeling (IbM) (38, 39, 51), and hybrid individual/continuum modeling (1). These models represent a discrete dynamic system whose behavior is completely specified in terms of a local relation, and are based on the idea that the complex behavior of a total system can be derived from simple local rules and interactions among the behaviors of elements. The stochastic discrete model compared with the deterministic model (such as ordinary or partial differential equations [ODE/PDE] based model) rather fits the modeling of a non-linear biological phenomenon, especially in the field of ecology, composed of many elements affecting each other, because the stochastic discrete model represents a self-organizing complex system.

**Fig. 5:**
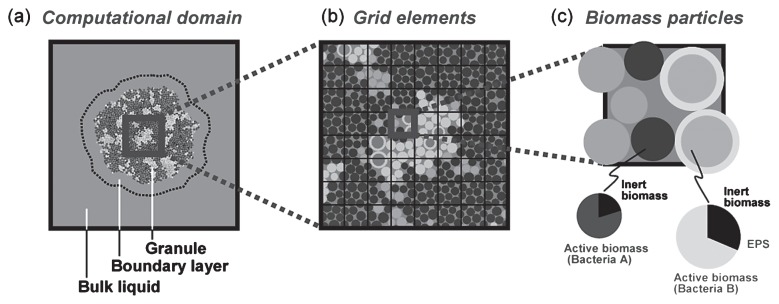
IbM model description (a) 2-D biofilm (granular type microbial aggregate is shown) in a square computational domain; (b) square grid elements discretizing the space, each containing several biomass particles; (c) individual biomass particles of different possible biomass types. All biomass particles within a single grid element experienced the same substrate concentrations (44).

Among them, IbM is appealing due to its more realistic representation of biomass division and spreading, which describes a biomass (a bacterial cell or bacterial biomass) as spherical particles with positions in space defined by continuous coordinates. Each biomass particle contains an active biomass of a single microbial type surrounded by an EPS capsule produced by the biomass within the particle. Each biomass particle grows and produces EPS. The biomass particles divide into two daughter particles when their size exceeds a critical size as a result of growth. Each “type (species)” has its own set of parameters. The spheres move when they are too close, resulting in biofilm spreading. In this model, the pressure that builds up due to biomass growth is relaxed by minimizing the overlap of spheres. Biomass-based IbM using larger biomass particles (10 to 20 μm in diameter) is more realistic for general use rather than treating a bacterial cell as a minimum unit, which sometimes requires too much computer power, while maintaining the moving or pushing principle for biomass redistribution. Fig. 5 describes IbM representing a microbial granule, a type of biofilm resulting from self-aggregation and growth as an example (42).

In the model, the calculations of soluble substrate profiles and biomass growth are separated. The biomass (particle) consumes the substrate in states calculated by solving the appropriate discrete diffusion/reaction equations (diffusion: usually according to Fick’s law; and reaction and growth: usually according to the Monod equation), but division, death and detachment are treated as stochastic events. In contrast, solute substrate profiles are solved by using differential equations based on mass balances that contain well-known biological reaction kinetics and mass transport terms. Such an approach acknowledges the enormous difference in size, and the time scale over which change can be observed between the biomass (cell) and substrate (molecule). More detailed descriptions of these models are shown in previous papers (51, 53, 67, 69).

### Combined approach with laboratory experimentation

A considerable number of studies have been conducted on the application of multi-dimensional models to analyze the various types of biofilm. They focus on various aims, for example, the analysis of detachment (6), the effect of EPS production on the community structure (69), the biofilm formed in membrane-aerated bioreactors (41), granular aggregate formations (42, 68) and biofouling (52) in the wastewater treatment process, the biofilm formed in microbial fuel cells (50), and biofilm development in the bioleaching process (47). Although there are various types of experimental techniques, which enable direct measurement of the microbial community, there have been limited numbers of studies on the experimental verification of multi-dimensional biofilm model predictions (41, 66). Furthermore, very few attempts have been made at evaluating the microbial eco-system in biofilms by combining the strategy of experimental and simulation analysis (42).

**Fig. 6:**
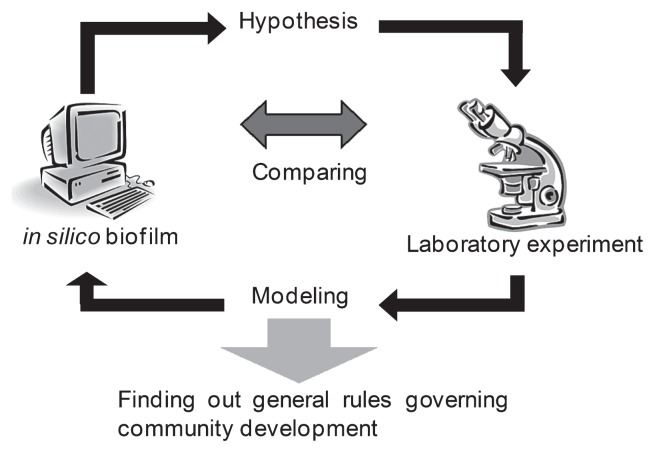
Schematic image featuring comparative analysis of the *in silico* approach and a laboratory experiment. The scheme of this analysis is 1) modeling of biofilm followed by computer simulation, 2) validation of the simulated data with the experimental data and verifying the hypothesis, and 3) reconstruction of the model followed by comparison with experimental data. This cycle continues to identify the essential factors for biofilm development.

This is probably because mathematical modeling and computer simulation are not familiar to most microbiologists. The models that have been developed so far are not sufficient in terms of simplicity and capability for general use. There remain several limitations: [i] computer power, although current computational efficiency is much higher than before; [ii] algorithms and modeling due to the problem of realistically not knowing all the components that exist in the biofilm to apply to the model; [iii] not knowing all the parameters precisely. Therefore, such models that represent complex systems of the microbial community include many simplified assumptions in order to capture a fraction of the biofilm features. This does not imply that *in silico* biofilm must be identical to “real biofilm.” Models can and should be improved through validation with laboratory experimental data and verification of a hypothesis (Fig. 6).

In explaining synthetic biology, a phrase often cited, Richard Feynman wrote, “What I cannot create, I do not understand.” Instead of constructing “real biofilm,” creating “virtual biofilm” through mathematical modeling and computational simulation, followed by comparative analysis with laboratory experimental data, would provide a better understanding of the general rules that govern the development of the biofilm eco-system. Therefore, the goal of this approach is to reconstruct biofilm with the minimum number of factors: that is to say, “What I understood, I can create.”

## Complex-systems biology for plasticity-diversity problems in ecosystem

Traditionally, population dynamics have often been adopted in theoretical studies of the ecosystem, in which each individual has no internal degree of freedom, and just the number of population of each species (or types) is involved. As already pointed out in the section “*Feedback between ecological and evolutionary dynamics: experimental study using planktonic microorganisms*”, such treatment has a limitations when discussing plasticity in phenotypic traits. In response to environmental changes, the phenotype is often changed, and this changeability is defined as plasticity. As a response to the environment is an essential feature of an organism, which influences the growth rate and the nature of interactions in population dynamics, phenotypic plasticity need to be taken into account seriously (19, 66). Here, if all the individuals belonging to the same genotype showed an identical response to the environmental change, then in considering plasticity, just the introduction of additional parameter(s) to the population dynamics would be sufficient, controlling the growth rate and species-to-species interaction of each species and depending on the plastic change of the phenotype.

However, experiments in bacteria and other organisms have elucidated that there is a large degree of phenotypic variation, even among isogenic individuals (3, 9, 11, 57). Hence, phenotypes are distributed even in isogenic individuals sharing the same environmental condition. An individual-based model with internal phenotypic variables is often postulated, as also referred to in the section “*In silico biofilm as a tool for investigation of microbial eco-system*”.

Even though such non-genetic phenotypic variation itself is not inherited by offspring, the degree of variance of phenotypes generally depends on genotypes, and thus can be inherited. Hence, it is interesting to study the possible relationship between isogenic phenotypic variance and evolution. Indeed, recent experimental, numerical, and theoretical studies have suggested that this phenotypic variation of isogenic individuals is proportional or positively correlated with the evolution speed of such phenotypes (26, 30, 55). This correlation may not be so surprising to physicists, as the proportionality between fluctuation and response against external change has been established in thermodynamics. By extending the fluctuation-response relationship in physics, it is proposed that phenotypic plasticity (changeability) is correlated with the variance of (isogenic) phenotypic fluctuation, as a consequence of the robustness of such phenotypes against external or internal perturbations (27, 29). Furthermore, recent numerical experiments have demonstrated the relevance of phenotypic plasticity by fluctuation to cope with environmental variations (28).

Although this isogenic phenotypic variance gives one quantitative measure of plasticity, further important steps are still missing to deal with the complexity of the ecosystem. The first is the discrete and discontinuous change in phenotypes. Environmental change often leads to such a change in phenotypes, as in polyphenism in the desert locust, Rotifer, Daphnia, and so forth (19). In terms of dynamical-systems theory, such change can be regarded as a result of bifurcation by representing a relevant environmental condition as a bifurcation parameter (such as temperature, population density of some species, and so forth). Indeed, in bifurcation, a slight change in a parameter can result in discontinuous change of the state value in the system in question. In this case, the plasticity concerns with how far the system’s parameter is from such a bifurcation point.

So far, we have discussed phenotypic plasticity against environmental change. To discuss the coexistence of diverse species under a given environmental condition, however, we need to take interaction among individuals into account, which is essential in a complex ecosystem that consists of a large number of individuals. Depending on the population of each species or type, interaction changes alter the environmental condition of each individual. This change in interaction may introduce a continuous or discrete change in the phenotype. A typical example is the change in phenotype depending on population density, as typically seen in polyphenism, depending on the density of the same species or of predator species.

The above two points, the change in phenotype by bifurcation and by interaction, are indeed integrated. Yomo and Kanko proposed ‘isologous diversification’, in which phenotypes of isogenic individuals are bifurcated into two (or more) groups, as a result of interaction with other individuals (26, 31, 32). Here, the interaction influences the developmental dynamics to shape the phenotype, and it works as a bifurcation parameter. Bifurcation to two (or more) phenotypic types occurs as a result of population change. When individuals of a novel distinct phenotype emerge, interaction between individuals of different phenotypes is changed, so that the interaction and diversification of types influence each other. In a certain condition, this mutual feedback leads to the stabilization of each distinct phenotype, as well as the population density of each type.

This isologous diversification was originally proposed for cell differentiation of a multicellular organism due to cell-cell interaction but, as a concept, it is generalized to any differentiation in the phenotypes of interacting organisms. Here, however, differentiation into types by isologous diversification is not yet speciation, since they still share the same genotype. However, when genetic change in the reproduction of each individual is included, non-genetic phenotypic differentiation is later fixed to genetic differentiation, and finally two groups with distinct phenotypes and genotypes are formed (which are also stable against sexual recombination, as hybrid sterility results). Hence robust sympatric speciation based on interactions was proposed (25, 32).

So far, isologous diversification has been applied only to speciation to two (or a few) types. To connect this concept with diversification in the ecosystem and phenotypic plasticity, we need to integrate the following processes that reinforce each other: [i] Introduction of a novel type of interaction enhances the phenotypic plasticity of certain individuals; [ii] With the increase in plasticity, groups of novel, distinct phenotypic types (and accordingly of distinct genotypes later) are generated; [iii] The increase in existing types of phenotypes introduces a new dimension in interaction. If this feedback progresses, mutual amplification among plasticity, interaction, and diversification will progress, so that a complex ecological system is shaped. Of course, whether this plasticity-interaction-diversification loop starts to reinforce itself generally depends on environmental conditions but, once started, the amplification will reach a stage that allows for the diversification of species even under sympatric conditions. Indeed, in this case, the effective environmental condition for each individual is influenced by the interaction with others, thus it depends on the population distribution of species and can change over 3 time.

The mechanism of the above reinforcement of the plasticity-interaction-diversification loop has not been established theoretically or experimentally. However, the experimental report on the coexistence of diverse types of *Escherichia coli* by Kashiwagi *et al.* (33) is suggestive. They found that bacterial communities increase the number of coexisting types when they are cultured under high density conditions, in which an increase in phenotypic plasticity was also suggested. Further experimental/theoretical studies of the plasticity-interaction-diversification loop will be important to understand its complexity and stability in ecosystems, including biofilms.

With this plasticity-interaction-diversification loop, a robust ecosystem is expected to be shaped, in which populations of diverse species with distinct phenotypes are maintained, while each phenotype remains stable. Indeed, one of the key concepts in complex-systems biology (26) is shaping the consistency between two hierarchical levels, *i.e.*, each individual element and the whole system consisting of such elements, by means of the interaction of elements with plasticity. It covers the consistency between molecule replication vs cellular reproduction, cell growth vs development of an organism, and genotypic vs phenotypic changes. Of course, consistency between replication of an individual organism vs sustainment of an ecosystem is another important issue, and we hope that concepts developed in complex-systems studies will be relevant for understanding the plasticity, robustness, and diversity of an ecosystem.

## Concluding remarks

The research introduced in this review shows how ecosystems become complex, what we know, what we do not know and what we can know at present, and additionally, what we should do and how it should be approached in the future. Indefinite species concepts and physiological elasticity/phenotypic variation of microbes increase the complexity of microbial ecosystems. Elements for modeling should be adjusted by each purpose and ecosystem, *e.g.*, metabolic group, phylogenetic species, physiological state or individual cell.

The prediction of interactions in microbial networks has been challenged by a comprehensive survey of microbiological processes in addition to mathematical analysis (11). In order to fully elucidate microbial ecosystems, however, mathematical approaches will be further combined with other analyses, *e.g.*, artificial neural networks (40), network theory (61), and systems analysis (63, 64). Systems analysis has been applied to several fields, *e.g.*, metabolic flux, economics and computer science in this century. Merry R. Buckley proposed “systems microbiology”, which treats the organism or community as a whole to create an integrated picture of how a microbial cell or community operates, in a report from the American Academy of Microbiology 2004 (Systems Microbiology: Beyond microbial genomics, http://academy.asm.org/). This may provide a conceptually different point of view from the previous perspective. As a limited example, the chaotic behavior of populations was found in the unpredictable behavior of microbes in a defined mixed culture (4). Consequently, socio-microbiology has been proposed as a keyword to draw the whole picture of the microbial ecosystem (23, 49).

As Charles Darwin proposed in “The Origin of Species” (7), diversification, adaptive evolution, and interspecies interactions could be produced by “laws”. How can we clarify these laws? Montoya and coworkers mentioned in their review article that a simple pattern representing ecological mechanisms can be defined for ecosystems (44). The microbial world was almost invisible before the recent development of molecular techniques and equipment. The microbial ecosystem may allow the establishment of ecological principles/rules and constitute appropriate tractable alternatives to ecosystems composed of long-lived macro-organisms that are harder to investigate. Researchers from a variety of disciplines, *e.g.*, biology, chemistry, geology, astronomy, mathematics, sociology, and so on, should join forces to obtain innovative achievements, which may completely change our understanding of the biosphere.
